# New-onset Parkinsonism as a Covid-19 infection sequela: A systematic review and meta-analysis

**DOI:** 10.1016/j.amsu.2022.104281

**Published:** 2022-08-08

**Authors:** Syed Sami Ali, Afshan Mumtaz, Mohammad Aadil Qamar, Sameer Saleem Tebha, Azma Parhin, Mehwish Butt, Mohammad Yasir Essar

**Affiliations:** a Medical College, Ziauddin University, Karachi, Pakistan; b Department of Neurology, Karachi Medical and Dental College, Karachi, Pakistan; c Department of Neurosurgery and Neurology, Jinnah Medical and Dental College, Karachi, Pakistan; d Department of Neurology, University of Washington, Seattle, WA, USA; e Kabul University of Medical Sciences, Kabul, Afghanistan

**Keywords:** COVID-19, SARS-CoV-2, Corona virus disease, Parkinsonism, Parkinson's disease

## Abstract

**Background:**

There remains a scarcity of literature regarding COVID-19 and its neurological sequelae. This study highlights Parkinsonism as a post-COVID-19 sequela and helps us understand a possible link between the two.

**Methods:**

A literature search covering relevant databases was conducted for studies reporting the development of Parkinsonism in patients recovering from COVID-19 infection. A quality assessment tool developed by The Joanna Briggs Institute Critical Appraisal tools for the assessment of case reports was utilized. Fisher's exact test was used to explore the factors associated with COVID-19 and Parkinsonism as its complication.

**Results:**

Ten studies were included in our study. The median age of patients was 60.0, with an interquartile range of 42.5–72.0. There were 8 males (61.5%) patients, and 53.8% of cases were reported to have at least one comorbidity. Cogwheel rigidity was the most common symptom of Parkinsonism in 11 patients. While the most standard treatment modality used was Levodopa in 76.9% of cases. Using the Fisher's Exact test, it was identified that 10 patients (76.9%) with bradykinesia made a full recovery.

**Conclusion:**

Despite presumed “recovery” from COVID-19, patients still face a wide range of neurological complications. One of these complications presenting as Parkinsonism requires health care professionals to be on the lookout for the long-term effects of COVID-19. Hence, our study provides information on the possible likely hood of a link between COVID-19 and the development of Parkinsonism as post-COVID neurological sequelae.

## Highlights

• There have been well-documented reports about the occurrence of parkinsonism after viral infections.
• 10 studies including 13 patients reported the development of parkinsonism in individuals who recovered from COVID-19 infection.
• The commonest symptom of parkinsonism reported in these individuals was cogwheel rigidity 84.6%.
• 53.8% of individuals fully recovered and had their symptoms resolved after treatment.

## 1 Introduction

Covid-19 (Corona Virus Disease) is a disease that is caused by a virus called SARS-CoV-2, which was discovered in December 2019 in Wuhan, China [1]. The virus belongs to the coronavirus family, the same family of the virus responsible for the epidemics of Middle East respiratory syndrome coronavirus (MERS-CoV) in 2012 and Severe acute respiratory syndrome coronavirus (SARS-CoV) in 2002 [2]. WHO(World Health Organization) declared it a global pandemic on March 11, 2020, and since then, it has been a public health care emergency [3]. As of April 29, 2022, there have been 509,531,232 confirmed cases of COVID-19, including 6,230,357 deaths reported to WHO since its onset [4]. The virus is reported to have a respiratory droplet and aerosol transmission-primarily through close contact when an infected person coughs or sneezes and an uninfected person inhales those liquid particles [5].

The disease presentation in COVID-19 varies in individuals, with some people presenting as asymptomatic/mild disease while others with a more severe illness and even death in some cases. Common symptoms include fever, cough, and shortness of breath [6]. Although Covid-19 primarily affects the respiratory system causing pneumonia, multi-organ failure and dysfunction have also been reported in more severe cases [7]. There is also evidence that SARS-CoV-2 can invade the neural tissue resulting in various neurological manifestations and complications [8,9]. Several theories regarding its route of entry and invasion into the CNS have been hypothesized. The first possible route for SARS-CoV-2 to enter the brain is across the blood-brain barrier (BBB). Several research reports show that the subunit S1 of SARS-CoV-2 S protein reaches the brain across the BBB [10]. In addition to these reports, it has also been proposed that there is a route of viral entry from the olfactory mucosa into the brain. In the olfactory mucosa, endothelial tissue and neural tissue are near each other. Hence the virus may use this to invade the brain, which can be confirmed by the presence of S proteins in the olfactory mucosa [11]. A review article also suggests a possibility of transsynaptic transmission of SARS-CoV-2 from the peripheral nerve [8]. Several patients of COVID-19 developed neurological symptoms such as headache, dizziness, hypogeusia, and neuralgia [[12], [13], [14]]. While on a more severe side of complications, there have been reports of covid-19 induced Parkinsonism. There have already been well-documented reports about Parkinsonism after viral infections [15]. The first link of viral Parkinsonism comes from the possible relationship between lethargic encephalitis and the Spanish flu of 1918 [16].

Parkinsonism is an umbrella term used to describe symptoms of Parkinson's disease, including bradykinesia, rigidity, tremor, and postural instability [17]. There have been several recent reports that support the notion that an increase in pro-inflammatory cytokines levels such as IL-6 and IL-1BETA may hasten the symptoms of Parkinson's. Since COVID-19 can result in a cytokine storm in many cases, it may be postulated that there can be an increased incidence of Parkinsonism in those who recover from the infection in years to come [18]. Thus, our study aims to bring all the reports of such COVID-induced parkinsonism cases together to help understand a plausible link between the two in a better way. We investigate the disease pattern, signs and symptoms, treatment modalities, and outcomes in patients with COVID-19 infection and the development of Parkinsonism in these patients as a post covid neurological sequelae.

## 2 Methods

The protocol of this review is registered with The International Prospective Register of Systematic Reviews (PROSPERO) [19]CRD42022325061. This review is fully compliant with the Preferred Reporting Items for Systematic Reviews and Meta-Analysis (PRISMA) 2020 statement and also in line with the PRISMA criteria [20].. The AMSTAR-2 [21] checklist was also used to assess this study which determined this study to be a high-quality review.

### 2.1 Search methods

An extensive literature review was conducted on seven major databases online: PubMed, Cochrane library, Google Scholar, ScienceDirect, China National Knowledge Infrastructure (CNKI) Database, MedRxiv, and BioRxiv, covering a timeline of January 1, 2020 to January 1, 2022. The following keywords were used to search: COVID-19 along with its derivatives and Parkinsonism along with its derivatives, as shown in S1 Table. No language restrictions were applied.

Case reports, Case series, Cross-sectional, Case-control, and Cohort studies aimed at reporting and determining the prevalence and association of Parkinsonism in a population infected with the COVID-19 virus were included in this study. We excluded review articles, opinion articles, and letters not presenting any original data. The Inclusion criteria were as follows: (1) We included covid-19 patients who developed Parkinsonism or Parkinson-like symptoms as a neurological complication after being infected with covid-19. A clinical diagnosis of Parkinsonism was made on the identification of a combination of some cardinal motor signs like bradykinesia, rigidity, tremor, and postural instability. (2) There was no gender or ethnic restriction. (3) A Positive Sars-CoV-2 rRt-PCR nasal swab of COVID-19. While the exclusion criteria were as follows: (1) Patients with a prior history or family history of Parkinson's disease. (2) Patients who might have any prior insult or injury to the brain that could lead to the development of Parkinson-like symptoms other than covid-19. (3) No family history of other neurodegenerative or neuropsychiatric disorders. (4) no history of exposure to drugs or neurotoxins like antidepressants or antipsychotic drugs may produce symptoms of Parkinsonism. (5) no history of metabolic or systematic disease like hypothyroidism that can lead to Parkinsonism. A third independent author resolved any discrepancies in selection.

### 2.2 Data extraction and analysis

The shortlisted articles were then extracted, independently and in duplicate, on a structured data form by two study authors. The information extracted from each study was as follows: the author's name, country, year of publication, type of study, study population, data source, age, number of patients with Covid-19, Positive Sars-CoV-2 rRt-PCR nasal swab, Covid-19 symptoms (including anosmia, cough, respiratory symptoms, GIT symptoms, fever, and myalgia), Parkinsonism symptoms (including anosmia, bradykinesia, cogwheel rigidity, tremor, hypokinetic rigid syndrome, saccades hypomimia and hypophonia), treatment modalities, any other comorbidities and outcomes for both, covid-19 and Parkinsonism, (full recovery, partial recovery, treatment ongoing or death).

SPSS Version 26 (Chicago, IL: IBM SPSS Statistics) was used to analyze the data. Quantitative variables were represented as median and interquartile ranges, whereas frequency and percentages were used for qualitative variables. Fisher's exact test was used to test for a significant relationship between the variables. The test results were considered statistically significant if the p-value was 0.05 or less.

### 2.3 Quality assessment

The authors evaluated individual study quality independently using The Joanna Briggs Institute Critical Appraisal tools for use in JBI Systematic reviews [22]. The study quality was scored out of 8 by the author, based on the patient's demographic description, patient history presentation, clinical condition presentation, diagnostic test results description, treatment description, post-intervention condition description, adverse events identification, and takeaway lessons. A study was good quality with a score of 6–8, fair quality with a score of 4–5, and poor quality with a score of <4.

## 3 Results

**Fig. 1:**
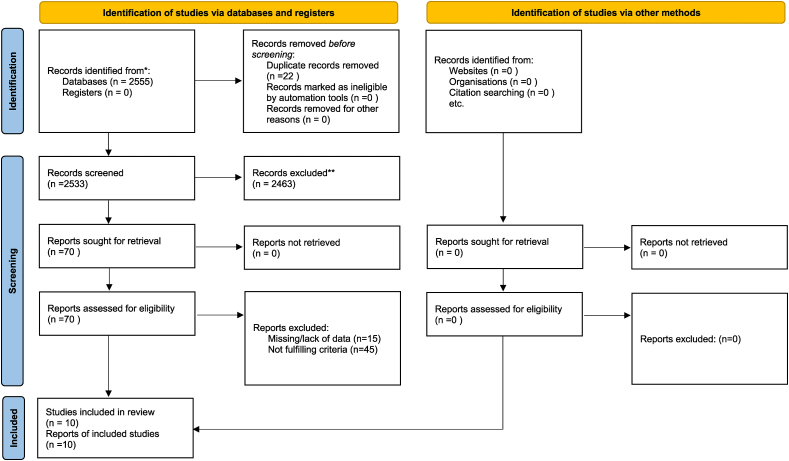
PRISMA 2020 flow diagram for new systematic reviews which included searches of databases, registers and other sources *Consider, if feasible to do so, reporting the number of records identified from each database or register searched (rather than the total number across all databases/registers). **If automation tools were used, indicate how many records were excluded by a human and how many were excluded by automation tools. *From:* Page MJ, McKenzie JE, Bossuyt PM, Boutron I, Hoffmann TC, Mulrow CD et al. The PRISMA 2020 statement: an updated guideline for reporting systematic reviews. BMJ 2021; 372:n71. https://doi.org/10.1136/bmj.n71. For more information, visit: http://www.prisma-statement.org/.

The comprehensive literature search yielded a total of 2555 studies from the databases. Overall, 10 studies were eligible according to our inclusion criteria [[23], [24], [25], [26], [27], [28], [29], [30], [31], [32]]. The characteristics of these 10 included studies are shown in Table 1. The PRISMA flow diagram presents an overview of the detailed selection process (Fig. 1).

**Table 1:** Characteristics of included studies (N = 10).

Study and year	Study design	country	Total Population	Age	Gender	Co-morbid	Covid-19 signs and symptoms	Covid-19 management	Parkinsonism signs and symptoms	Radiological investigations		Parkinson's management	Recovery from parkinsonism symptoms
Mikhal E Cohen 2020	Case report	Israel	1	45	Male	Hypertension and asthma	Respiratory symptoms, anosmia, cough, fatigue, and myalgia	None.	Bradykinesia, cogwheel rigidity, tremor, hypomimia, hypophonia	F-DOPA-PET scan: decreases F-FDOPA uptake in both putamen more apparent on left side	Dopaminergic therapy, anticholinergic, corticosteroids		Partial recovery
Mendez Guerrero 2020	Case report	Spain	1	58	Male	Hypertension and dyslipidaemia	Respiratory symptoms, anosmia, cough, GIT symptoms, fever, and fatigue	Steroids, protease inhibitor, mechanical 02 ventilation	Anosmia, cogwheel rigidity, postural tremor, resting tremor, myoclonus, hypokinetic rigid syndrome, vertical saccades and hypomimia	DaT scan: bilateral decrease in presynaptic dopamine uptake. EEG: diffuse mild and reactive slowing without any asymmetries or epileptiform discharges.	none		Partial recovery
Ingrid Faber 2020	case report	Brazil	1	35	Female	None.	Respirator symptoms, anosmia, cough, GIT symptoms, fever, and myalgia,	None.	Anosmia, bradykinesia, cogwheel rigidity, hypokinetic rigid syndrome, hypomimia, hypophonia and slow and hypometric saccades.	DaT scan: decreased dopamine transporter activity on left putamen	Levodopa and benserazide		Full recovery
Mauro Morassi 2021	Case report	Italy	2	1)70 2)73	Female	1)Hypertension 2)Hypertension and diabetes	1)Respirator symptoms, anosmia, cough, and fever. 2)Fever.	1)02 supplementation, Hydroxychloroquine, and protease inhibitor. 2)O2 supplementation	1)Bradykinesia, cogwheel rigidity, myoclonus, hypomimia and hypophonia. 2)Cogwheel rigidity, tremor, hypokinetic rigid syndrome and hypomimia.	1) EEG: bilateral theta-delta slowing with bitemporal epileptiform discharges. FDG-PET: diffuse cortical hypo-metabolism MRI: ventricular enlargement 2)EEG: theta slowing with sharp waves over the right fronto-temporal region. FDG-PET: diffuse cortical hypo-metabolism.	Levodopa and corticosteroid		Partial recovery
Conor Fearon 2021	Case report	N/A	1	46	Male	None.	Respirator symptoms, fever, and cough	Mechanical ventilation	Bradykinesia, cogwheel rigidity, hypophonia and hypomimia.	Brain CT and MRI: oedema in globus pallidus.	Levodopa		Partial recovery
Nazire Belgin 2021	Case report	N/A	1	72	Male	Hypertension, diabetes, and peripheral artery disease.	Respirator symptoms, cough, and fever	Mechanical ventilation	Bradykinesia, cogwheel rigidity, tremor.	None.	Levodopa		Full recovery
Abhijith Rao 2022	Case series	N/A	3	1)722) 66 3)74	Male	1)None. 2)Hypertension, diabetes, and seizures. 3)None.	1)Respirator symptoms, anosmia, cough, and fever. 2)Respiratory symptoms and cough. 3)Respirator symptoms.	1)Steroids and remdesivir. 2)Steroids and oxygen supplementation. 3)None.	1)Anosmia, bradykinesia, cogwheel, and rigidity. 2)Bradykinesia, hypophonia and cogwheel rigidity. 3)Bradykinesia, hypophonia and cogwheel rigidity.	1)None. 2)MRI: gliosis in bilateral temporal lobes. 3)MRI: ischemic changes in paraventricular white matter.	Levodopa		Full recovery
Devjit Roy 2021	Case report	USA	1	60	Male	Hypertension, diabetes, and hypercholesterolemia	Respirator symptoms and cough	Mechanical ventilation	Hypokinetic rigid syndrome and hypophonia	MRI: basal ganglia and corona radiata stroke.	Levodopa		Partial recovery
Ayele 2021	Case report	Ethiopia	1	35	Female	None.	Anosmia and fever	Steroids and acyclovir	Bradykinesia, cogwheel rigidity, tremor, resting tremor, hypomimia and hypophonia	MRI: Flair hyperintense lesion.	Levodopa		Full recovery
Pietro Tiraboschi 2021	Case report	Italy	1	40	Female	None.	Respiratory symptoms, anosmia fever and fatigue	Mechanical ventilation	Bradykinesia, postural tremor, and myoclonus	EEG: bilateral slow waves with epileptiform discharges FDG-PET: Increased metabolism in the mesial temporal lobes and subthalamic nuclei	None.		Full recovery

EEG: Electroencephalography, F-DOPA-PET: F-Fluorodopa-positron emission tomography, FDG-PET: Fludeoxyglucose-positron emission tomography, CT scan: computerized tomography, DaT scan: dopamine active transport, MRI: magnetic resonance imaging, N/A: Not available, GI symptoms: gastrointestinal symptoms.

In terms of study type, of the included 10 studies, 9 (90%) were case reports, and only 1 study was case series (10%).

### 3.1 Demographics and epidemiology

13 patients with a median age of 60.0 and IQR (42.5–72.0) were included in this study. The gender of study cases was 61.5% males and 38.5% females. Of the 13 cases, 7 (53.8%) reported at least one comorbidity. Hypertension is the most prominent one with a frequency of 7(53.8%), then diabetes with a frequency of 4 (30.8%), and then asthma with a frequency of 1(7.7%), as shown in Table 1.

### 3.2 Clinical features, management, and outcome of COVID-19 infection

The commonest symptom during COVID-19 infection was respiratory symptoms (11 patients, 84.6%) which were followed by cough (9 patients, 69.2%) and fever (9 patients, 69.2%) while myalgia was the lowest presenting symptom (2 patients,15.4%). Regarding management, the most administered treatment, according to our study, was mechanical ventilation (5 patients, 38.5%). At the same time, three patients (23%) received no treatment for COVID-19 infection. The two outcomes reported in our study were either death or full recovery (refer to Table 2).

**Table 2:** Covid-19 signs and symptoms, management, and outcome.

Signs and symptoms	N(%)
Respiratory symptoms	11 (84.6)
Anosmia/hyposmia/dysgeusia	7 (53.8)
Cough	9 (69.2)
GI symptoms	3 (23.1)
Fever	9 (69.2)
Fatigue	3 (23.1)
Myalgia	2 (15.4)
Treatment	
Steroids	4 (30.8)
Hydroxychloroquine	2 (15.4)
Acyclovir	1 (7.7)
Remdesivir	1 (7.7)
Protease inhibitor	2 (15.4)
Mechanical ventilation	5 (38.5)
Oxygen supplementation	3 (23.1)
Outcome	
Hospitalisation	12 (92.3)
Full recovery	11 (84.6)
Death	1 (7.7)

### 3.3 Clinical features, management, lab investigations, and outcome of Covid-19-induced Parkinsonism

All patients developed symptoms of Parkinsonism as post-COVID-19 neurological sequelae, but it took each patient a different number of days to develop Parkinson's symptoms after contracting the COVID-19 infection, as shown in Table 1. The commonest symptom of Parkinsonism was cogwheel rigidity (11 patients,84.6%) and bradykinesia (10 patients,76.9%). While the least common symptoms were postural tremors (2 patients,15.4%) and saccades (2 patients, 15.4%). The commonest management received were Levodopa (10 patients,76.9%) and corticosteroid (3 patients,23.1%). While Benserazide (1 patient,7.7%), Dopaminergic therapy (1 patient, 7.7%), and Anticholinergic (1 patient,7.7%) were the least administered treatment. (7 patients,53.8%) patients fully recovered, and their symptoms fully resolved after treatment, while (6 patients,46.2%) patients partially recovered, and symptoms persisted.

A set of different laboratory tests and investigations were run on the patients. The most common abnormal findings were reported on magnetic resonance imaging (MRI) which was abnormal in 6(46.2%) cases, followed by electroencephalography (EEG) and fluorodeoxyglucose (FDG)-positron emission tomography (PET), both of which showed abnormal findings in 3(23.1%) cases (For the rest of the findings, refer to Table 3).

**Table 3:** Parkinsonism signs and symptoms, radiological findings, management, and outcome.

Signs and symptoms		N (%)
Anosmia/hyposmia		3 (23.1)
Bradykinesia		10 (76.9)
Cogwheel rigidity		11 (84.6)
Tremor		4 (30.8)
Postural tremor		2 (15.4)
Resting tremor		3 (23.1)
Myoclonus		3 (23.1)
Hypokinetic rigid syndrome		6 (46.2)
Saccades		2 (15.4)
Hypomimia		7 (53.8)
Hypophonia		8 (61.5)
Radiological findings		
EEG findings	Normal	1 (7.7)
	abnormal	3 (23.1)
	N/A	9 (69.2)
F-DOPA PET scan		
	abnormal	1 (7.7)
	N/A	12 (92.3)
FDG-PET scan	Normal	1 (7.7)
	abnormal	3 (23.1)
	N/A	8 (61.5)
Brain CT scan	Normal	4 (30.8)
	abnormal	1 (7.7)
	N/A	8 (61.5)
DaT scan	abnormal	2 (15.4)
	N/A	11 (84.6)
MRI findings	Normal	6 (46.2)
	abnormal	6 (46.2)
	N/A	1 (7.7)
Treatment		
Levodopa		10 (76.9)
Benserazide		1 (7.7)
Dopaminergic therapy		1 (7.7)
Anticholinergic therapy		1 (7.7)
Corticosteroid		3 (23.1)
Recovery	Partial recovery	6 (46.2)
	Full recovery	7 (53.8)

EEG: Electroencephalography, F-DOPA-PET: F-Fluorodopa-positron emission tomography, FDG-PET: Fludeoxyglucose-positron emission tomography, CT scan: computerized tomography, DaT scan: dopamine active transport, MRI: magnetic resonance imaging, N/A: not available.

The odds between symptomatic presentation in COVID-19 and Parkinsonism, management, comorbid, and outcomes were measured using fisher's exact test at a p-value less than equal to 0.05. It was identified that patients who had anosmia in covid-19 infection didn't have diabetes as a comorbid (53.8%) at a p-value = 0.021. The rest of the findings are shown in Table 4.

**Table 4:** Fisher’ Exact test.

variable				p-value
anosmia present in covid-19 infection:		Diabetes as a comorbid present: n (%)		0.021
		YES	NO	
	yes	0 (0.0%)	7 (53.8%)	
	NO	4 (30.8%)	2 (15.4%)	
Fatigue present: n (%)		Postural tremor present: n (%)		0.038
		YES	NO	
	YES	2 (15.4%)	1 (7.7%)	
	NO	0 (0.0%)	10 (76.9%)	
Fatigue present: n (%)		Levodopa used as treatment: n (%)		0.03
		YES	NO	
	YES	0 (0.0%)	3 (23.1%)	
	NO	10 (76.9%)	0 (0.0%)	
Hyroxychloroquine used for treatment: n (%)		Protease inhibitor used for treatment: n (%)		0.013
		YES	NO	
	YES	2 (15.4%)	0 (0.0%)	
	NO	2 (15.4%)	11 (84.6%)	
hydroxychloroquine used for treatment		Myoclonus present: n (%)		0.038
		YES	NO	
	YES	2 (15.4%)	0 (0.0%)	
	NO	3 (23.1%)	10 (76.9%)	
protease inhibitor used for treatment: N (%)		Myoclonus present: n (%)		0.038
		YES	NO	
	YES	2 (15.4%)	0 (0.0%)	
	NO	1 (7.7%)	10 (76.9%)	
full recovery: N (%)		Bradykinesia present: n (%)		0.038
		YES	NO	
	YES	10 (76.9%)	1 (7.7%)	
	NO	0 (0.0%)	2 (15.4%)	
postural tremor present: N (%)		Myoclonus present: n (%)		0.038
		YES	NO	
	YES	2 (15.4%)	0 (0.0%)	
	NO	1 (7.7%)	10 (76.9%)	

### 3.4 Quality assessment of included studies

Three out of 10 studies were identified as good quality, while seven were of fair quality, as shown in Table S2. Studies were primarily downgraded for unclear patient's demographic description [27], incomplete patient's history presentation [24,25,[27], [28], [29], [30], [31], [32]], inadequate diagnostic test results description [28,30], unclear treatment description [25,27,28,32], incomplete post-intervention condition description [[23], [24], [25],^29^], no proper adverse events identification [[23], [24], [25], [26], [27], [28],[30], [31], [32]] and not enough takeaway lessons [23]. The most common cause for downgrading studies was no proper adverse events (harms) or unanticipated events being identified and described, which raised concerns if the treatment/intervention/drugs used has more benefit than risks and the if the benefits outweigh the risks.

## 4 Discussion

Both the developing and developed countries are still battling the spread of COVID-19. A major concern that needs to be addressed is what doctors are now calling the “Long COVID.” [14].

There have been several studies and enough data collection on complications in patients recovering from COVID-19 infection after 3 months, 6-months, and 1 year. Complications range from neurodegenerative diseases to impaired cognitive mental function [[33], [34], [35]]. Similar reviews on COVID-19 have previously been conducted with a broader agenda of tackling all the neurological complications, but none of the available literature focused on the grave dangers of long-term complications of Parkinsonism specifically. Hence our review provides extensive information regarding Parkinsonism associated with COVID-19 infection in terms of analyzing all the clinical features and presentations.

The median time since the development of parkinsonism symptoms in patients in our study was 14 days (IQR = 14.5 days). In a prospective observational study of 135 COVID-19 patients, new onset of neurological diseases was diagnosed after a 3-month follow-up who previously had not reported any such neurological manifestations, with Parkinsonism being one of them [33]. In this study, bradykinesia, tremor, and rigidity were reported in n = 7(5%), n = 13(10%), and n = 3(2%), respectively, all of these being cardinal neurological presentations in Parkinsonism. These findings are in line with our study, where n = 10(76.9%) patients presented with bradykinesia, n = 4(30.8%) presented with tremors, and n = 11(84.6%) with rigidity. The primary neurological manifestation was anosmia/hyposmia in this observational study with a prevalence rate of 45% while in our study, anosmia was reported in 23.1% of patients. After the 3-month study, a 1-year follow-up study was carried out on these patients however only 76 of these 135 patients completed the follow-up. In addition, 5 patients were evaluated for 1-year follow-up and added to this study. Of these 81 new-onset parkinsonism was reported in two more patients [35]. In this study, bradykinesia was reported in n = 5(6%), tremors reported in n = 2(2%), and rigidity reported in n = 5(6%) patients. In another cohort study of 236,379 patients diagnosed with COVID-19, Parkinsonism was diagnosed in 0.11% of the patients after a 6-month follow-up [34]. These findings show how it makes each individual a different number of days to develop signs of parkinsonism and in some cases, it may even take up to a year. Hence, this highlights the importance of extensive follow-up that is required in patients recovering from COVID-19.

According to our study, anosmia/hyposmia was one of the most frequently occurring symptoms in COVID-19 patients. Anosmia is also one of the most common non-motor features of Parkinson's disease [36]. This highlights the possibility of cerebral involvement in COVID-19, and it suggests that SARS-CoV-2 has the potential to invade the brain through olfactory pathways [37]. Several different hypotheses have been made on probable routes of entry for virus invasion into the CNS. One of the key pathways for neurotropic invasion is the olfactory pathway. The unique anatomical organization of the olfactory bulb and olfactory nerves in the nasal cavity and the forebrain can act as a channel for viruses to enter the brain compartments. SARS-CoV-2 is suggested to reach these brain structures using this pathway in the early stages of infection to cause inflammation and degenerative processes [38].

Another observation made in our review was that almost all patients with bradykinesia after COVID-19 showed a full recovery from parkinsonian symptoms. Showing that most of the cases showed a good response to Levodopa or a dopamine agonist. The DaT scans of both the patients (19,20) showing decreased presynaptic dopamine uptake further suggest a possible virus invasion into the CNS. Angiotensin-converting enzyme-2 (ACE-2), which is widely expressed in various tissues, including the brain, could be another possible route of entry for the virus to enter the host brain cells and cause neuronal injury. SARS-CoV-2 could interact with ACE-2 in the capillary endothelium and cause blood-brain barrier destruction, which would promote virus entry into the CNS. Evidence shows mRNA is present in the brain of infected individuals of SARS-CoV, which also interacts with ACE-2. The high similarity between SARS-CoV and SARS-CoV-2 suggests an abundance of ACE-2 in brain tissues promoting the entry of SARS-CoV-2 into the CNS and subsequently causing neuroinvasion [39].

Pinto and colleagues examined over 700 patients with comorbidities including hypertension, cardiac diseases, and diabetes having severe COVID disease. In these patients, they observed a high expression of ACE-2 in epithelial tissues and theorized that it could be one of the reasons for the high prevalence of anosmia in patients with COVID-19. The extracellular domain of ACE-2 is the cellular receptor of SARS-CoV-2 spike proteins, which interacts with triggering endocytosis of the virus. The interaction seems to be increased in patients suffering from comorbidities [40]. Even in our study 7 out of 13 patients also had some co-comorbidity with diabetes in 4 cases. However, Pinto's findings do not concur with our data as we noted that 53.8% of patients with anosmia did not have diabetes as a comorbid at a p value = 0.021. Hence more research needs to be done before anyone can speculate about the impact diabetes, as a comorbid, can have on increasing or decreasing the chances of anosmia in COVID-19 patients.

### 4.1 Strengths and limitations

The major strengths of this study were that no language restrictions were applied and key reference lists were additionally screened for more studies, however, we did not find any studies that could meet our eligibility criteria.

A major limitation of this study was the small sample size that was analyzed. There were not enough data and case reports available to quantify the associated risk of developing Parkinsonism as a post-COVID neurological complication hence a more extensive analysis could not be done. Another drawback of the small sample size is that we couldn't generalize our findings to the whole population. Only with time, as more case reports will be available in the future, a better relationship between the two can be understood.

## 5 Conclusion

To our knowledge, this study is the first study that highlights Parkinsonism as major neurological sequelae of COVID-19 infection. The findings of this study raise an alarming concern regarding the potential dangers that COVID-19 could present to the world in the future and how there needs to be more emphasis on what doctors have been calling the “Long COVID”. However, a more thorough analysis needs to be done before a causal link between the two can be made. This would require more extensive data collection and long-term follow-up of the patients presenting with Parkinsonism as a post-COVID complication.

## Ethical approval

This study did not need any ethical approval being a systematic review and meta-analysis of publicly available data.

## Source of funding for your research

This study had no sources of funding

## Author contribution

SSA conceptualized the study design and objectives. AM, MAQ, and SSA drafted the study protocol, and conducted the literature search, study screening, selection, and data extraction. AM, MAQ, and SSA designed the data collection instrument, collected data, and carried out data analysis. SSA, AM, MAQ, SST, AP, and MB drafted the initial manuscript. AM, MAQ,SST, and MYE critically reviewed and revised the final manuscript. SSA is the guarantor and critically reviewed the manuscript. All authors approved the final manuscript as submitted for publication.

## Registration of research studies

1. Name of the registry: International Prospective Register of Systematic Reviews (PROSPERO).

2. The unique identifying number or registration ID: CRD42022325061.

3. Hyperlink to your specific registration (must be publicly accessible and will be checked): https://www.crd.york.ac.uk/PROSPERO/display_record.php?RecordID=325061.

## Guarantor

Syed Sami Ali is the guarantor.

## Consent

Not applicable.

## Provenance and peer review

Not commissioned, externally peer-reviewed.

## Data availability statement

Data is available upon request to the corresponding author.

## Declaration of competing interest

None declared.
