# Supplementary material for: New-onset Parkinsonism as a Covid-19 infection sequela: A systematic review and meta-analysis
Source: Ann Med Surg (Lond). 2022 Aug 8;80:104281. doi: 10.1016/j.amsu.2022.104281 (PMC9359766; doi:10.1016/j.amsu.2022.104281)
Supplement: Multimedia component 1 [file mmc1.docx]

S1 Table. Search strategy for PUBMED

| 1. | "COVID-19"[Mesh] OR "COVID 19"[tw] OR "COVID-19 Virus Disease"[tw] OR "Coronavirus Disease 2019"[tw] OR "SARS Coronavirus 2 Infection"[tw] OR "SARS-CoV-2 Infection"[tw] OR "SARS-CoV-2 Infections"[tw] OR "COVID-19 Virus"[tw] OR "Wuhan Coronavirus"[tw] OR "SARS Coronavirus 2"[tw] OR "Severe Acute Respiratory Syndrome Coronavirus 2"[tw] |
| --- | --- |
| 2. | "Parkinson Disease"[Mesh] OR Parkinson[tw] OR "Lewy Body Parkinson's Disease"[tw] OR "Parkinson's Disease"[tw] OR "Primary Parkinsonism"[tw] OR “Secondary Parkinsonism” [tw] OR Parkinsonism[tw] |
| 3. | 1 AND 2 |

Search strategy for Google Scholar

| 1. | As a post covid-19 sequelae “Parkinsonism” |
| --- | --- |

Search strategy for ScienceDirect

| 1. | Covid-19 AND Parkinsonism |
| --- | --- |

Search strategy for Cnki

| 1. | covid-19 OR "COVID-19 Virus Disease" OR "SARS-CoV-2 Infection" OR "SARS Coronavirus 2 Infection" AND Parkinsonism |
| --- | --- |

Search strategy for Cochrane Library

| 1. | "COVID 19" OR "COVID-19 Virus Disease" OR "Coronavirus Disease 2019" OR "SARS Coronavirus 2 Infection" OR "SARS-CoV-2 Infection" OR "SARS-CoV-2 Infections" OR "COVID-19 Virus" OR "Wuhan Coronavirus" OR "SARS Coronavirus 2" OR "Severe Acute Respiratory Syndrome Coronavirus 2" |
| --- | --- |
| 2. | Parkinson OR "Lewy Body Parkinson's Disease" OR "Parkinson's Disease" OR "Primary Parkinsonism" OR “Secondary Parkinsonism” OR Parkinsonism |
| 3. | 1 AND 2 |

Search strategy for Medrxiv and Biorxiv

| 1. | COVID-19 AND Parkinsonism |
| --- | --- |
