# Supplementary material for: New-onset Parkinsonism as a Covid-19 infection sequela: A systematic review and meta-analysis
Source: Ann Med Surg (Lond). 2022 Aug 8;80:104281. doi: 10.1016/j.amsu.2022.104281 (PMC9359766; doi:10.1016/j.amsu.2022.104281)
Supplement: Multimedia component 2 [file mmc2.docx]

| Serial number | First author | Were the patient’s demographics characteristics clearly described? | Was the patient’s history clearly described and presented as a timeline? | Was the current clinical condition of the patient on presentation clearly described? | Were diagnostic tests or assessment methods and the results clearly described? | Was the intervention(s) or treatment procedure(s) clearly described? | Was the post-intervention clinical condition clearly described? | Were adverse events (harms) or unanticipated events identified and described? | Does the case report provide takeaway lessons? | Total | Level of evidence |
| --- | --- | --- | --- | --- | --- | --- | --- | --- | --- | --- | --- |
| 1 | Mikhal E Cohen | yes | yes | yes | yes | yes | unclear | no | no | 5 | fair |
| 2 | Mendez Guerrero | yes | unclear | yes | yes | yes | unclear | no | yes | 5 | fair |
| 3 | Ingrid Faber | yes | unclear | yes | yes | unclear | Unclean | no | yes | 4 | fair |
| 4 | Mauro Morassi | yes | yes | yes | yes | yes | yes | no | yes | 7 | good |
| 5 | Conor Fearon | unclear | no | yes | yes | unclear | yes | no | yes | 4 | fair |
| 6 | Nazire Belgin | yes | unclear | yes | no | no | yes | no | yes | 4 | fair |
| 7 | Abhijith Rao | yes | unclear | yes | yes | yes | Unclean | yes | yes | 6 | Good |
| 8 | Devjit Roy | yes | no | yes | no | yes | yes | no | yes | 5 | Fair |
| 9 | Ayele | yes | unclear | yes | yes | yes | yes | no | yes | 6 | Good |
| 10 | Pietro Tiraboschi | yes | unclear | yes | yes | unclear | yes | no | yes | 5 | Fair |

Answers: Yes, No, Unclear, Not/Applicable

Decisions about scoring were discussed by all reviewers before critical appraisal assessment, and a study was characterised as having a high risk of bias when it reached a “yes” score of up to 49% (bad level of evidence), moderate when 50% to 69% (fair level of evidence) and low when greater than 70% (good level of evidence)
